# Perspective: Telehealth – beyond legislation and regulation

**DOI:** 10.1177/20503121221143223

**Published:** 2023-01-10

**Authors:** Khamis Al-Alawy, Immanuel Azaad Moonesar

**Affiliations:** Mohammed Bin Rashid School of Government, Dubai, UAE

**Keywords:** Legislation, regulation, telehealth, telemedicine, health innovation

## Abstract

The World Health Organization describes health innovation as developing new or improved systems, policies, products, technologies, services or delivery approaches that improve health and well-being, specifically of vulnerable people. The study’s objectives were to (a) explore the legislative and regulatory journey of telehealth across the Organisation for Economic Co-operation and Development and non-Organisation for Economic Co-operation and Development countries and (b) provide recommendations to strengthen health system performance. We reviewed information sources for Organisation for Economic Co-operation and Development and non-Organisation for Economic Co-operation and Development countries opportunistically, including government and medical board publications, media coverage and peer-reviewed papers, to provide a perspective on the legislative and regulatory telehealth journey. Our review of countries suggests that legislation and regulation remain essential for governance, accountability and assuring that healthcare professionals and technologies are safe and secure. However, there was no uniform approach to telehealth legislation and regulation, and the precautionary approach was observed in some countries. Different strategies appear to have been adopted for telehealth implementation. There is a need to go beyond legislation and regulation to strengthen health system performance and assure the future success of telehealth services. Health system decision makers should work with health system stakeholders to strategise and plan for telehealth services as it will have implications on the future delivery of healthcare services and the health system. Further research is needed to explore how policy frameworks may support innovations in healthcare, such as telehealth.

## Introduction

### Telehealth today

The World Health Organization (WHO) describes health innovation as the development of new or improved systems, policies, products, technologies, services or delivery approaches that improve health and well-being, specifically, vulnerable people.^1^ Health innovation may, for example, respond to an unmet health need by spearheading new approaches to improve people’s health. The fruition of health innovations within the scope of telehealth has undoubtedly broadened the spectrum and potential for healthcare service delivery. Telehealth has become integrated into clinical practice in ways that stretch the imagination with increasing acceptance and pace. Telehealth is no longer viewed as the mere interaction between two healthcare professionals or between the patient and the physician at a distance but rather it encompasses a myriad of features and services.^2^ For example, a large part of telehealth lies in the back-office transfer of diagnostic data and reports from one site to another for specialist interpretation and reporting or synchronous monitoring of Intensive Care Unit (ICU) data.^3,4^ In recent years, telehealth has gained greater acceptance among the public as a viable alternative for face-to-face consultations and the management of chronic diseases. As seen in Table 1, it has also prevailed in preventing COVID-19 transmission globally.^5,6^

**Table 1.** Telehealth and telemedicine initiatives and actions in the era of COVID-19.^5,6^

Countries	First confirmed COVID-19 case date	Telehealth usage during COVID-19	Telehealth regulations before COVID-19	Telemedicine initiatives^a^									New telemedicine policies/process/systems by MoH or authority website/documents
				Tele-stroke	Teleradiology	Tele-ICU	Tele-mental health	Telepathology	Telesurgery	Telemonitoring	Telepharmacy	Teleconsultation	
China	17th November 2019	√	√	√	√	x	x	√	√	√	√	√	√
Thailand	13th January 2020	√	x	√	√	√	√	√	√	√	√	√	√
Japan	14th January 2020	√	√	√	√	√	√	√	√	√	√	√	√
USA	19th January 2020	√	√	√	√	√	√	√	√	√	√	√	√
South Korea	20th January 2020	√	√	x	√	√	x	x	x	√	x	x	x
Singapore	23rd January 2020	√	√	√	√	x	x	√	√	√	√	√	x
France	24th January 2020	√	√	√	√	√	√	√	√	√	√	√	√
Australia	25th January 2020	√	√	√	√	√	√	√	x	√	√	√	√
Canada	25th January 2020	√	√	√	√	√	√	√	√	√	√	√	x
Germany	27th January 2020	√	√	√	√	√	√	√	√	√	√	√	√
UAE	29th January 2020	√	√	x	√	√	√	√	√	√	√	√	√
India	30th January 2020	√	x	√	√	√	x	√	√	√	√	√	√
Spain	31st January 2020	√	x	√	√	√	x	√	√	√	√	√	x
Italy	31st January 2020	√	√	√	√	√	√	√	√	√	√	√	√
Egypt	14th February 2020	√	x	x	√	x	x	√	x	x	x	x	x
Iran	19th February 2020	√	x	x	√	x	√	x	x	x	x	√	x
Brazil	26th February 2020	√	√	√	√	√	√	√	√	√	√	√	√
UK	28th February 2020	√	√	√	√	√	√	√	x	√	√	x	√
South Africa	1st March 2020	√	√	x	√	x	√	x	x	x	x	x	√
Jordan	3rd March 2020	√	x	x	x	x	x	x	x	x	x	√	x
Sweden	11th March 2020	√	√	√	√	x	√	√	x	x	x	x	x

√:Yes, sufficient evidence; x: No sufficient evidence/data not available; MoH: Ministry of Health/Authorities.

a As of end of December 2020.

### Literature review and context

**Figure 1.**
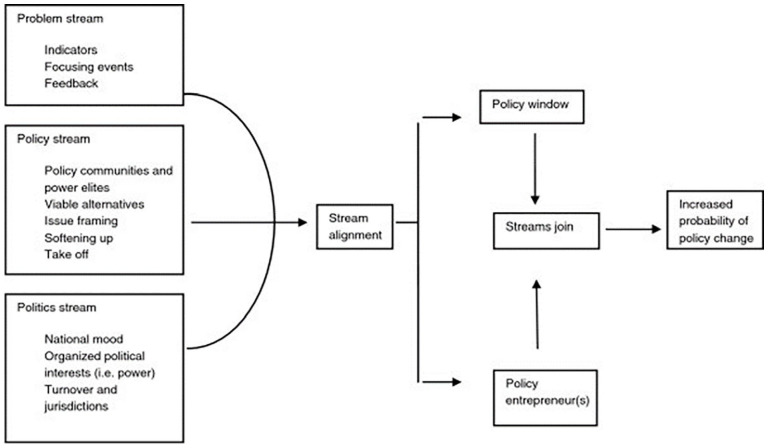
Kingdon’s multiple streams approach.^11^

While innovations such as telehealth often outpace legislation and regulation, their importance in assuring if the health system is fit for purpose should not be overlooked. There are several reasons why telehealth has been subjected to legislation and regulation. *Firstly*, healthcare services and technologies are entwined with health service provision and reimbursement; thus, they need to be governed.^7^ *Secondly*, telehealth has been accepted as part of medical practice; therefore, healthcare professionals remain accountable for telehealth practice.^8,9^ *Thirdly*, there are confidentiality and data protection implications for telehealth practices; thus, technologies used in the healthcare setting must meet the requirements of patient safety and assure data protection and security.^10^ *Lastly*, telehealth is seen as a vehicle to address health system constraints of cost, access and quality. Government institutions and medical boards are responsible for issuing legislation and regulation but also bear the responsiblity to ensure health system contraints are addressed. Kingdon’s Multiple Streams Approach (MSA), seen in Figure 1, has been used to explain how and why competing policy agendas may come to fruition within the political system.^11^ The MSA suggests that three streams (problem, policy, political) work individually and in parallel to bring about policy window and policy entrepreneurship, which creates an opportunity for policy change or development amidst other competing policy agendas. In this context, MSA may explain why telehealth legislation and regulation may or may not occur within the health system. MSA has been widely used across several disciplines and it aids empirical research; however, it has also been criticised due to its inapplicability and lack of comprehensiveness in different policy contexts.^12,13^ As legislation and regulation play an important role in enabling health innovations, we explored the telehealth journey across different health systems and provided recommendations on strengthening health system performance.

## Methods

We reviewed information sources for Organisation for Economic Co-operation and Development (OECD) and non-OECD countries opportunistically, including government and medical board publications, media coverage and peer-reviewed papers, to provide a perspective on the legislative and regulatory telehealth journey. This is relevant to explore a phenomenon, event or policy in a real-life context and can assist in understanding current gaps, future needs or approaches that may be particularly useful for reflective practice. Based on our review of available information sources, we opportunistically sampled eight OECD and non-OECD countries that were leading the global share of the telehealth market by region.^14–17^ Countries selected include France, the United Kingdom, Australia, Sweden, Singapore, South Africa, UAE and Brazil. We reviewed available laws, regulations, policies, standards, guidelines, medical board guidance, media coverage and peer-reviewed papers in each country until 2021. These information sources are useful to capture the telehealth journey of each country and highlight the unintended consequences. The review excluded organisational arrangements and those within government departments as these are likely to vary considerably from one country to another and are difficult to retrieve. The MSA was used as a guide for reviewing the legislative and regulatory telehealth journey for each country. A benchmark of eight countries against key criteria required for telehealth was also undertaken and summarised in a table. The criteria included the presence of law, policy approach, dedicated strategy, government funding, health insurance coverage, integration with EMR, teleprescribing, requirement to have an established relationship with the patient, requirement for accreditation and requirement to report on key performance indicators.

## Telehealth journey

### France

French Law formally defined telemedicine^18^ and included teleconsulting, tele-expertise, telemonitoring, teleassistance and medical regulation.^19^ Following this, a bidding process for telehealth providers took place, and eight telehealth experiments (adults in the community, institutionalised patients, neonates, inmates, surgical patients and complex pathological interpretation) were funded across the region by the Paris Regional Health Agency – Agence Régionale de Santé Ile-de-France.^20^ To assure the conditions for deployment of telemedicine were in place, a conventional agreement was concluded between health insurance representatives for private doctors on 14 June 2018, with amendment no. 6 to the national convention of 25 August 2016.^21^ On 15 September 2018, telemedicine entered the common law for health insurance reimbursement.^22^ On 10 February 2019, the law was enacted, and physicians were allowed to offer remote teleconsultation and tele-expertise for the same cost as face-to-face consultations. Conditions for reimbursement include video call, adoption of care pathways and the requirement for patients to be known by the physician or healthcare professional in the past 12 months.^23^ In 2020, Decree 2020-227 allowed 100% payment for all persons exposed to COVID-19 from any physician during wave 1^24^. To date, France has conducted several evaluations to inform policy decision-making. For example, in 2019, a report was issued on the findings for phase 1 implementation and highlighted the general acceptance by doctors and citizens.^24^ In contrast, field workers and health professionals expressed their reservations on using teleconsultation for high-risk patients and their preference for face-to-face consultation for accurate diagnosis and treatment.

### United Kingdom

The emergence of telehealth in the United Kingdom dates back to the 1990s; however, its fruition was hindered due to a lack of funding, training and ongoing concerns on medical liability protection and how insurance coverage would work across the four countries. In 2000, the Department of Health attempted to revive telehealth, but there was a lack of progress on digitisation. In the past decade, the establishment of the Care Quality Commission, Clinical Commissioning Groups, NHSX, NHS Digital, Medical Device Regulations, Standards and Guidelines for Digital Technology, Digital Strategies and an ongoing commitment towards research and start-ups have catalysed telehealth.^25–28^ However, several challenges remain, such as implementing the Good Data Protection Regulations (GDPR), the Medicines and Healthcare Products Regulatory Agency regulation for digital health apps and cross-border working following Brexit. Integration of software and medical devices, machine learning and ethical artificial intelligence also remains at the forefront of legal and regulatory discussions. Before COVID-19, there was much controversy among NHS-funded General Practitioners (GP), Clinical Commissioning Groups and NHS London. GPs (independent contractors) felt virtual telehealth providers were pinching their patients, which meant several GPs were losing patients with minor illnesses. This meant that GPs were left with a cohort of complex patients making it difficult to achieve financial sustainability. On the other hand, patient waiting time and satisfaction scores have improved since the commissioning of virtual telehealth providers.^29^

### Australia

In 1975 Australia introduced universal healthcare (Medicare), and the state was tasked to manage healthcare services while the Commonwealth was given oversight for its Pharmacy Benefits Scheme.^30^ The Australian Health Practitioner Regulation Agency (AHPRA) is Australia’s primary legislative and regulatory entity supported by 14 national boards; thus, new directives are often issued by AHPRA or the national boards.^31^ The evolution of telehealth in Australia was founded primarily to serve rural areas. Despite the issuance of several legislative Acts more than a decade ago, progress towards telehealth was slow due to the lack of clarity on reimbursement and medical liability for misdiagnosis, data transfer and the use of sub-optimal technology. Also, there were restrictions on telehealth practice. For example, e-prescribing beyond the healthcare practitioner’s locality was not permitted.^32^ In recent years, Australia has undergone six stages of telehealth reform. In response to healthcare practitioners’ calls and public demand to ensure access to healthcare services during COVID-19, the Australian government issued interim provisions and subsidies for telehealth through its Medical Benefits Schedule and stage 7 reform. This was supported by the national boards, which later issued guidelines for their practitioners and allied health professionals.^33,34^ Reimbursement was subsequently granted for chronic disease management, management of pregnant women, mental health services and the elderly. While it was not initially a prerequisite, it is now mandatory for patients to be known by their physician, raising concerns about widening health inequalities.^35^ Exceptions to the mandate include under 12-year-olds and patients who are homeless.

### Sweden

Telemedicine in Sweden started in 1915 when the first known trial for telemedicine was done through remote reading of electrocardiogram signals at the Lund University Campus.^36^ In 1970, telepathology and teleradiology were introduced, and in 1990, telehealth was catalysed across several branches of medicine. In 1999, 60% of hospitals in Sweden were engaged in some form of telemedicine activity, and a further 15% were planning its use.^37^ Approximately 54% of telemedicine applications were used for specialist consultation (13% between paramedics and hospitals and 10% for rounds, with the majority utilised for teleradiology). In 2001, the Swedish Ministry of Health (MoH) assigned a working group to look at telemedicine and published a report in 2002 (‘Vård ITiden’) that discussed the strategies and measures needed to broaden the scope and utilisation of telemedicine.^38^ Subsequently, the National Board of Health and Welfare (Socialstyrelsen), a government agency under the MoH, developed guidance on care and treatment using digital health services (Digital Care on Overarching Principles for Treatment for Care – Digitala vårdtjänster Övergripande principer för vård och behandling).^39^ More recently, other regulations that apply to digital health and healthcare information technology were introduced, namely, the General Data Protection Regulation governs (2016/679) (‘GDPR’) and the Swedish Act, with supplementary provisions to the European Union’s Data Protection Regulation – SFS2018:218.^40^ Telemedicine in Sweden includes digital health technologies: virtual care, robotics, wearables, virtual assistants, mobile health apps, software as medical devices, artificial intelligence as a service, Internet of Things (IoT) and connected devices, 3D printing/bioprinting and natural language processing.^41^ The transformation of primary care services through telehealth has led to controversy over current regulation due to the rising costs of digitisation and how it will affect access and fair cost to services within the 21 county councils.^42^

### Singapore

Singapore’s telehealth is regulated through various guidelines and ethical codes of the Singapore Medical Council (SMC).^43^ The last revision of the SMC Ethical Code and Ethical Guidelines (ECEG) was issued in 2002 to reflect the changes in medical practice and communication technologies, including telemedicine and remote surgery. In 2015, the MoH issued National Telemedicine Guidelines focusing on four key domains: clinical standards and outcomes, human resources, organisational and technology, and equipment.^44^ The following year, the Health Products (Licensing of Retail Pharmacies) Regulations and Telepharmacy Guidelines were enforced to monitor ‘the provision of retail pharmacy services by a qualified pharmacist at a retail pharmacy, through a computer, or video or audio link’.^45^ The Singapore Health Sciences Authority issued regulatory guidelines on telehealth products, including medical devices.^46^ The regulatory approach within the guideline adopted risk-based and confidence-based regulation principles. Subsequently, the Singapore Dental Council’s ECEG were updated for the inclusiveness of remote consultations and continuity of care.^47^ However, there is no overarching law or regulation governing telehealth in Singapore. There are plans for telehealth to be regulated through the Healthcare Services Act, which will be implemented in three phases. The regulation of telemedicine is being planned toward the end of 2021 by the Singaporean MoH, with a view to license doctors and dentists in 2023.^48^ In preparation for the enactment of telemedicine, the MoH has commenced telemedicine e-training for doctors and dentists. Telemedicine presents a trade-off between convenience and high-quality care; thus, it is acknowledged that while telemedicine may benefit population health, there are gaps in knowledge and evidence base.

### South Africa

The national system for telemedicine in South Africa began in 1998 with a series of pilots across six provinces.^49^ The primary objective was to provide medical care and interventions to underserved communities and improve communication and links between healthcare facilities and underdeveloped urban areas. There have been eight associated laws, regulations and guidelines issued for telemedicine: National Health Act 61 of 2003, Health Professions Act 56 of 1974, the Medicines and Related Substances Act and Amendment Act 14 of 2015, Consumer Protection Act 68 of 2008, Electronic Communications and Transactions Act 25 of 2002, Protection of Personal Information Act 4 of 2013, Promotion of Access to Information Act 2 of 2002, and Health Professions Council of South Africa (HPCSA) Telemedicine Guidelines (2020).^50^ To assure public protection, the Allied Health Professions Act (Unprofessional conduct: telemedicine) was introduced in November 2011, which prohibited medical procedures such as homeopathy, naturopathy, phytotherapy, chiropractic and osteopathy, therapeutic aromatherapy, therapeutic reflexology, therapeutic massage therapy and acupuncture, Chinese medicine and Unani-Tibb. These professions were deemed not appropriate for the practice of telemedicine. In August 2014, the HPCSA was tasked with regulating health professions. HPCSA developed and published the General Ethical Guidelines for Good Practice in Telemedicine.^51^ In March 2020, COVID-19 Guidelines, Telehealth and Telemedicine were regulated as an extraordinary policy decision to permit the practice of telehealth and telemedicine during the pandemic.^52^ However, there were challenges in implementation due to infrastructure and availability of physicians in rural areas.

### United Arab Emirates

The UAE health authorities have spearheaded telehealth and telemedicine services in the region since 2013. The issuance of Federal Law Decree No. (4) of 2016 on Medical Liability and Addendum to Cabinet Decree No. (40) of 2019 on the Executive Regulations of Federal Medical Liability Law No. (4) of 2016 Terms and Rules for Telehealth Service was seen as critical milestones for telehealth.^53^ This was furthered by the announcement of the 50-year Charter, Article (5) of 2019, which set out the right for every citizen to access a doctor 24/7 through digital applications.^54^ The initiative targeted nationals and residents. In 2019, Federal Decree No. (2) for Information Communication Technology in the Healthcare Sector was announced.^55^ The Decree set out the specific requirements for health information exchange, data security, storage and confidentiality. The incremental approach towards digitisation in the UAE paved the way for the leading regulators to build upon the existing strengths and opportunities. For example, the Department of Health (DoH) in Abu Dhabi was the first to implement a dedicated telemedicine centre for its residents and a bespoke reimbursement model with the main insurer.^56^ Dubai Healthcare City Regulatory Authority was the first to regulate telehealth platform in the free trade zone and was the first to develop bespoke licensing processes.^57^ Dubai Health Authority was the first to forge public and private partnerships working through its ‘doctor for every citizen initiative’.^58^ The Ministry of Health and Prevention (MoHaP) was the first to lead on tele-ICU for remote critical care in addition to a diverse range of virtual clinics and specialised services during COVID-19 to include general practice, nursing, pharmacy, cardiology, paediatrics, internal medicine, gynaecology, mental health, nutrition and physiotherapy.^59^ The emergence of COVID-19 catalysed the use of digital platforms for telehealth and led to new regulatory workstreams to assure the reliability and security of telehealth platforms.

### Brazil

The emergence of telehealth in Brazil dated to the late 1970s when medical schools needed to adopt tele-education.^60^ From 1988 to 2019, approximately 79 telehealth regulations were reported to have been issued by the federal government and 31 rules from the national council of health professionals; for example, in 2000, the MoH developed a coalition for telemedicine (Ordinance No. 494/2000); in 2002, telehealth regulation was issued by the Professional Board of Medicine (1643/2002); in 2007, the Brazilian telehealth pilot project was launched (Ordinance No. 35/2007); in 2011, Telehealth Brazil Network Program (Ordinance No. 2546/2011) and the provision of telehealth services within the public health system were issued (Ministry of Health Resolution No. 2549/2011).^61^ This was a key turning point for telemedicine to transition from the private to the public sector. In 2014, the Federal Council of Medicine (CFM) introduced a regulation for teleradiology to allow the transfer of data and images (CFM No. 2107/2014); in 2018, new medical procedures and therapies to ensure patients’ safety, convenience and efficiency were introduced (No. 12 842), and the online consultations, tele-diagnostics, telesurgeries, tele-triage, telemonitoring and telediagnosis were approved for people in remote areas (CFM No. 2,227). In 2018, the General Data Protection Law (Federal Law No. 13 709/18 or LGPD) was also issued to match the European Union GDPR.^61,62^ More recently, telepathology regulation was introduced to allow the transfer of data and images (CFM No. 2264/2019), and regulation for tele-dentistry (No. 226/2020) was issued to monitor treatments already in progress and regulation for COVID-19 (No. 13 989/2020).^63^ Telehealth in Brazil includes mobile health (mHealth), Health Information Technology, wearable devices, telemedicine, personalised medicine, machine learning and artificial intelligence and have been steadily growing over the years, particularly among high-income earners with private health insurance. Telehealth has become a prominent part of primary care, psychiatry and family medicine; however, the approval of workplace and social media platforms for telehealth has raised concerns regarding confidentiality, data security and medical record keeping. There has also been a lack of clarity given the abundance of regulations issued since 1988 and standardised processes for designing and establishing a defined regulatory framework for telemedicine.

### Benchmark of countries

A benchmark of the eight countries shows uniformity in a top-down policy approach; however, different strategies appear to have been adopted for implementation (Table 2). For example, several countries do not have a dedicated telehealth strategy, mandate accreditation, and provider performance metrics for reporting. It remains unclear if bespoke approaches (or lack of) towards implementation were influenced by evidence, politics, infrastructure, funding and medical and social perspective, among other contextual factors.

**Table 2.** Benchmark of countries – telehealth and telemedicine initiatives by each country as of March 2021.

No.	Countries		Availability of a law for telehealth/telemedicine	Policy approach	Availability of a dedicated strategy for telehealth	Availability of govt funding stream to support telehealth start-ups	Inclusion in health insurance coverage	Full Integration of EMR across public and private sector	Teleprescribing allowed	Requirement of an established relationship with the patient	Mandatory accreditation for telehealth	Mandatory reporting on key performance indicators
1.	France	OECD	√	Top-down	x	√	√	x	√	√	x	x
2.	UK		X	Top-down	x	√	√	X	√	x	x	√
3.	Australia		x	Top-down	x	x	√	x	√	√	x	x
4.	Sweden		x	Top-down	√	x	√	x	√	x	x	x
5	Singapore	Non-OECD	x	Top-down	x	√	√	x	√	x	x	√
6.	South Africa		√	Top-down	x	x	√	x	√	x	x	x
7.	UAE		√	Top-down	x	√	√	√	√	x	√	√
8.	Brazil		√	Top-down	x	√	√	x	√	√	√	x

√: Yes, sufficient evidence; x: No sufficient evidence/data not available.

### Status quo

Based on the countries reviewed, there does not appear to be a uniform approach towards telehealth legislation and regulation. This presents challenges in formulating a blueprint and learning from best practices. There is a tendency for non-OECD countries to adopt more laws and regulations and take on a more scattered approach compared to OECD countries. For example, in incremental order, Singapore has adopted and updated several guidelines and regulations across different professional institutions without a telehealth law planned for 2022. South Africa adopted eight laws and regulations in 1998, followed by further measures in 2014 and during COVID-19. In the UAE, several federal laws, local regulations and standards were adopted before the bespoke telehealth, and information communication technology laws were enacted in 2019. In Brazil, there were approximately 79 legislative and regulatory reference points. In France, several assurance steps were taken after the telemedicine law was enacted in 2010, including launching the ETAPES project in 2014 to support telehealth financing in nine regions and across the entire country. At the local level (Paris), eight pilot projects were established to determine efficiency (task shifting), effectiveness (patient management) and acceptability (public and healthcare professionals) in different settings.^20^ The combination of these projects supported 70% insurance coverage for telehealth in 2018, chronic disease coverage in 2019 and 100% payment during the first wave of COVID-19.^21–23^

### Quid pro quo

The review of countries suggests that legislation and regulation are needed to govern and support innovations entering the market and drive efficiency, such as reducing primary care visits for repeat prescriptions, among many other factors. However, a top-down approach towards policy development may have unintended consequences as it may drive bespoke improvements (quick fixes) that are inconsequential to health system priorities or exclude certain forms of innovations, thus limiting the opportunity to solve systemic issues. For example, the enablement of teleprescribing has led to many platforms limiting the opportunity to develop a system-wide integrated telepharmacy solution for specialised services such as paediatrics, internal medicine or haematology.^63^ An alternative approach may include a bottom-up approach that entails joined-up working between patients, healthcare professionals, innovators and regulators to inform legislation. For example, there may be a better way to transfer data between patients and physicians. Patients could be empowered to select and approve specific and time-limited data for telediagnosis and telemonitoring. This would reduce the risk of unauthorised data access, data sharing or breaches in data confidentiality.

### Beyond legislation and regulation

While legislation and regulation are seen as key to driving innovations in healthcare, there are several factors that influence health system improvement. *First*, performance improvement is predicated on values, vision, mission and strategy, which are essential building blocks to instil confidence among investors and innovators and foster sustainable collaborations.^64^ *Second*, grants to support trial and error are limited, cumbersome and often competitive. Small- to medium-sized enterprises struggle to compete with larger entities and fold at the bidding processes’ first hurdle. The few who succeed may struggle to navigate through legislative, regulatory and implementation processes, which translates into unplanned delays and hidden costs. *Third*, due to COVID-19, telehealth is now perceived as an essential requirement for business continuity among healthcare providers, yet payment models have limited flexibility to support a strong business case for innovative change or business continuity.^65,66^ In addition, opportunities for the efficient management of services and service redesign at times of crisis are compounded by innovations that continue to work in silo with limited integration capabilities due to limited payment models and mandate.^67^ *Fourth*, care models within a single health system of public and private providers may vary considerably, risking the alignment of priorities. Business viability competes with patient care and continuity, making it difficult to decipher the evidence base, the type of care needed, when it is needed and how much care is needed.^68,69^ Health systems that embed evidence-based practice are arguably more capable of deciphering innovations that add value and those that are not cost-effective.^70,71^ *Lastly*, evaluation is often forgotten or lacking due to a lack of prioritisation, planning or workforce capacity.^72,73^ By mandate of the law, governments are the custodians of the health system and are accountable to assure that the legislative and regulatory mandates are successfully implemented to improve health system performance.^70,72^ In this context, decision-makers should go beyond the norms of more legislation and regulation and direct efforts towards the future success of telehealth (Table 3).

**Table 3.** What can government do to assure the future success of telehealth.

No.	Recommended action	Examples
1.	Support and adopt new ways of thinking and conceptualisation	• Make use of actionable words can affect investment bids, grants, strategic planning and job descriptions • Creating a compelling narrative to attract entrepreneurs, healthcare providers and patients • Visualise how collaborative transformation will improve the patients daily life
2.	Invest and support trial and error	• Document failures to help steer workstreams and pipeline projects • Make learning project based and ensure the learning is shared • Fund public and private innovation incubators to promote and boost health system knowledge and promote cross-sector collaboration
3.	Develop flexible payment models	• Payment models should indicate what, how, when and whom to pay • Make tiered payment models for telehealth possible: • Tier 1 payment, for example, could be based on duration, complexity, skill set/speciality or volume • Tier 2 payment requires more complex data collecting and monitoring systems for payment by results, high-risk groups or impact on health system efficiency, effectiveness or quality
4.	Support the adoption of broader perspectives with stakeholders	• Decision-makers should identify systemic concerns, the relevant elements and how telehealth can effect change • Minimise payment weighting on micro level improvements as they are likely to lead to little benefits along the pipeline and often insufficient to balance healthcare costs or enhance patient outcomes • Mandate collaborative, cost-effective, methodical and sustainable workstreams to benefit the health system
5.	Track macro and micro change	• Macro change includes changes in prescribing patterns, wait times, patient experience, adverse occurrences, integration or adopted of approved care models. Micro change includes all activities that lead to macro change, for example, mandatory training, staff roles and responsibilities, popup warnings (red flags) within a telehealth platform to promote physician compliance or digitising documentation to improve coding accuracy for reimbursement Set key performance measures to provide a real-time understanding of the micro and macro change

### Theoretical implications

Reflecting on the MSA, the countries reviewed provide an insight as to how telehealth policy issues may come onto the policy agenda. For example, telehealth was an ongoing policy problem for rural areas in Australia and became a priority with the emergence of COVID-19. On the other hand, MSA may not apply in certain instances or may be limited in understanding why the policy is prioritised, for example, a policy review cycle or due to the natural maturation or strengthening of existing policy. Telehealth existed in the United Kingdom and Brazil for many years, but several reasons led to its maturation, including the creation of institutions, the development of technology and public acceptance of digital health and mobile technology. Walt and Gilson provide a theoretical framework to understand the context, content, actors and policy process. Thus, opportunities to understand contextual challenges and the process should not be overlooked.^74^ On the other hand, the Centre for Disease Control and Prevention Policy Development Cycle (PDC) provides a practical approach to understanding policy development, including problem identification, policy analysis, strategy and policy development, policy enactment, policy implementation, evaluation and stakeholder engagement and education.^75^ PDC may be better suited for countries seeking policy revision or development.

### Limitations and directions for future research

The perspective has limitations. Our searches were limited to publicly available information from government institutions and medical boards; thus, obtaining greater insight within the organisation or across departments was not possible. Due to resource and time constraints, we could not review more than eight countries, limiting the opportunity to provide a perspective from other regulatory jurisdictions or explore different perspectives, for example, low-middle income countries. The review excludes stakeholder and public opinions, which may offer different perspectives on the journey and acceptance of telehealth. Finally, further research is needed to explore how policy frameworks may support innovations in healthcare, such as telehealth.

### Policy implications

They are several policy implications that can be considered. *Firstly*, policy design and implementation should ensure that the key components of e-health (the umbrella) are strengthened to assure the success of telehealth. For example, the absence of information and decision support systems such as electronic medical records hinder the opportunity for physicians to get on board with telehealth and is likely to affect high-quality care and patient satisfaction. *Secondly*, reimbursement should be sufficient to improve access and coverage, particularly where access to specialist care is limited. A monitoring mechanism should be implemented to detect inappropriate and overprescribing of telehealth practices. *Thirdly*, regulators should define the boundaries for telehealth practices as not all symptoms and diseases can be managed through telehealth alone. This will reduce costs and medical errors associated with inappropriate telehealth practices and referrals. *Fourthly*, healthcare practitioners should receive formal training on telehealth practices to assure clinical competence and public confidence. In areas of high risk, there is a need for medical training and specialisation to be certified, such as tele-ICU and telesurgery. *Fifthly*, theoretical frameworks provide insight into the policy and political environment, but policy development per se is a complex and muddy process that is not always linear or rational. The fact that telehealth has manifested to where it is today is commendable, but many policy questions remain unanswered. For example, where should telehealth sit within the health system (upstream, downstream, in the middle or across the entire continuity of care spectrum), how cost saving can be realised, and what forms of telehealth practices are safe and evidence based. *Lastly*, policy design, implementation and evaluation should be pursued, for example, differences in legislative approaches and types of telehealth services that are superior. Health system stakeholders should consider these facets in the context of telehealth because they are likely to play a key part as medicine, technology and patient expectations continue to evolve and entwine.

## Conclusions

Despite its importance, there is a need to go beyond legislation and regulation to strengthen health system performance and assure the future success of telehealth services. Health system decision makers should work with health system stakeholders to strategise and plan for telehealth services as it will have implications on the future delivery of healthcare services and the health system. Further research is needed to explore how policy frameworks may support innovations in healthcare, such as telehealth.

## Supplemental Material

sj-docx-3-smo-10.1177_20503121221143223 – Supplemental material for Perspective: Telehealth – beyond legislation and regulation Click here for additional data file. Supplemental material, sj-docx-3-smo-10.1177_20503121221143223 for Perspective: Telehealth – beyond legislation and regulation by Khamis Al-Alawy and Immanuel Azaad Moonesar in SAGE Open Medicine

sj-jpg-1-smo-10.1177_20503121221143223 – Supplemental material for Perspective: Telehealth – beyond legislation and regulation Click here for additional data file. Supplemental material, sj-jpg-1-smo-10.1177_20503121221143223 for Perspective: Telehealth – beyond legislation and regulation by Khamis Al-Alawy and Immanuel Azaad Moonesar in SAGE Open Medicine

sj-jpg-2-smo-10.1177_20503121221143223 – Supplemental material for Perspective: Telehealth – beyond legislation and regulation Click here for additional data file. Supplemental material, sj-jpg-2-smo-10.1177_20503121221143223 for Perspective: Telehealth – beyond legislation and regulation by Khamis Al-Alawy and Immanuel Azaad Moonesar in SAGE Open Medicine
