# Supplementary material for: Perspective: Telehealth – beyond legislation and regulation
Source: SAGE Open Med. 2023 Jan 10;11:20503121221143223. doi: 10.1177/20503121221143223 (PMC9834783; doi:10.1177/20503121221143223)
Supplement: sj-docx-3-smo-10.1177_20503121221143223 – Supplemental material for Perspective: Telehealth – beyond legislation and regulation [file sj-docx-3-smo-10.1177_20503121221143223.docx]

## **What Do We Already Known on This Topic?**

- Innovation in healthcare may respond to unmet health needs by spearheading new approaches to improve people's health, particularly those that are most vulnerable;
- The fruition of medical and health technologies have undoubtedly broadened the spectrum and potential for healthcare service delivery and opportunities to control cost within the health system; and
- Due to the rapid change in technology and medicine, telehealth is no longer viewed as the mere interaction between two healthcare professionals or between the patient and the physician at a distance but rather a myriad of features and services.

## **How Does Your Research Contribute to The Field?**

- Legislation and regulation remain essential for governance, accountability and assuring healthcare professionals and technologies are safe and secure however; there is no uniform approach among the countries reviewed which presents challenges in formulating a blueprint and learning from best practices;
- The precautionary principle is seen among some countries, while others have catalysed telehealth due to the Covid-19;
- Non-OECD countries appear to have adopted more laws and regulations and when compared to OECD countries; and
- A top-down policy approach is common among the countries reviewed and there is a lack of uniformity in the implementation of telehealth.

**What are your research’s implications towards theory, practice, or policy?**

- A top-down approach toward policy development may have unintended consequences when driving bespoke improvements (quick fixes) that are inconsequential to health system priorities, and exclude certain forms of innovations, thus limiting the opportunity to solve health system wide issues such as cost containment;
- Despite its importance, there is a need to go beyond legislation and regulation to strengthen health system performance and assure the future success of telehealth services; and
- The government should work with health system stakeholders to strategize and plan for telehealth services as it will have implications on the future delivery of healthcare services and the health system.
