# Supplementary figures and images for: Perspective: Telehealth – beyond legislation and regulation
Source: SAGE Open Med. 2023 Jan 10;11:20503121221143223. doi: 10.1177/20503121221143223 (PMC9834783; doi:10.1177/20503121221143223)

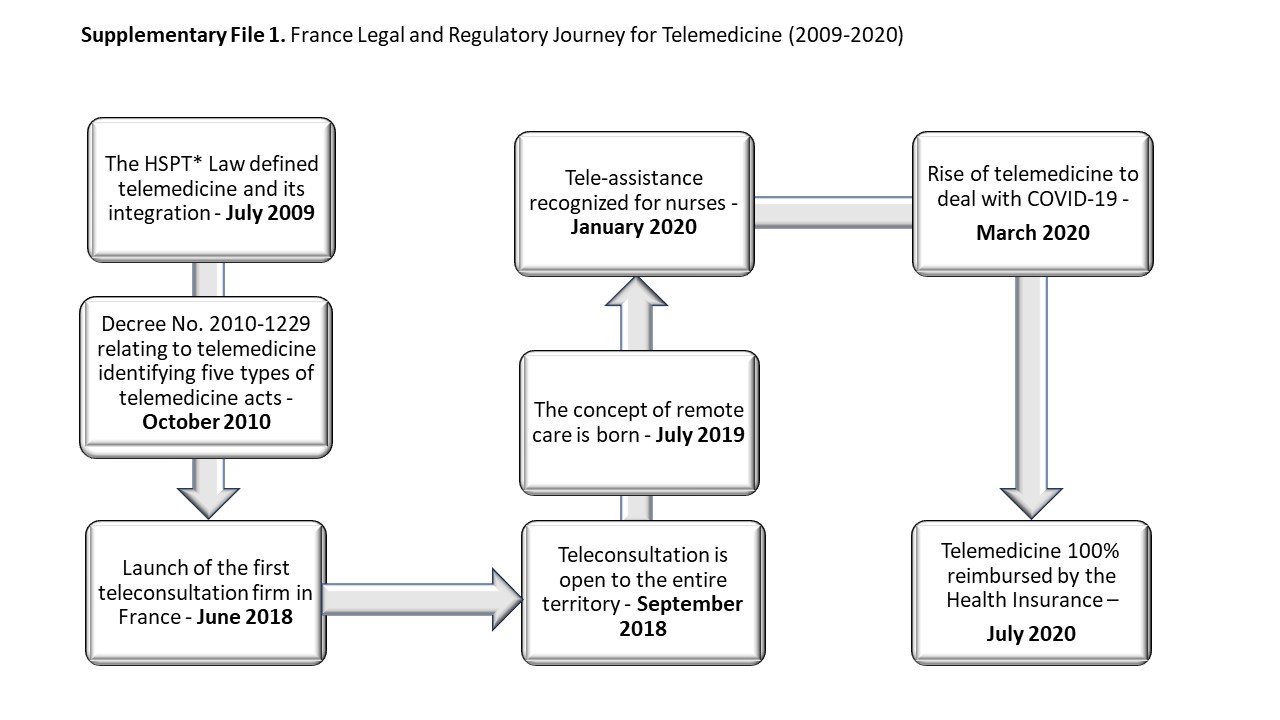

Supplement: sj-jpg-1-smo-10.1177_20503121221143223 – Supplemental material for Perspective: Telehealth – beyond legislation and regulation [file sj-jpg-1-smo-10.1177_20503121221143223.jpg]

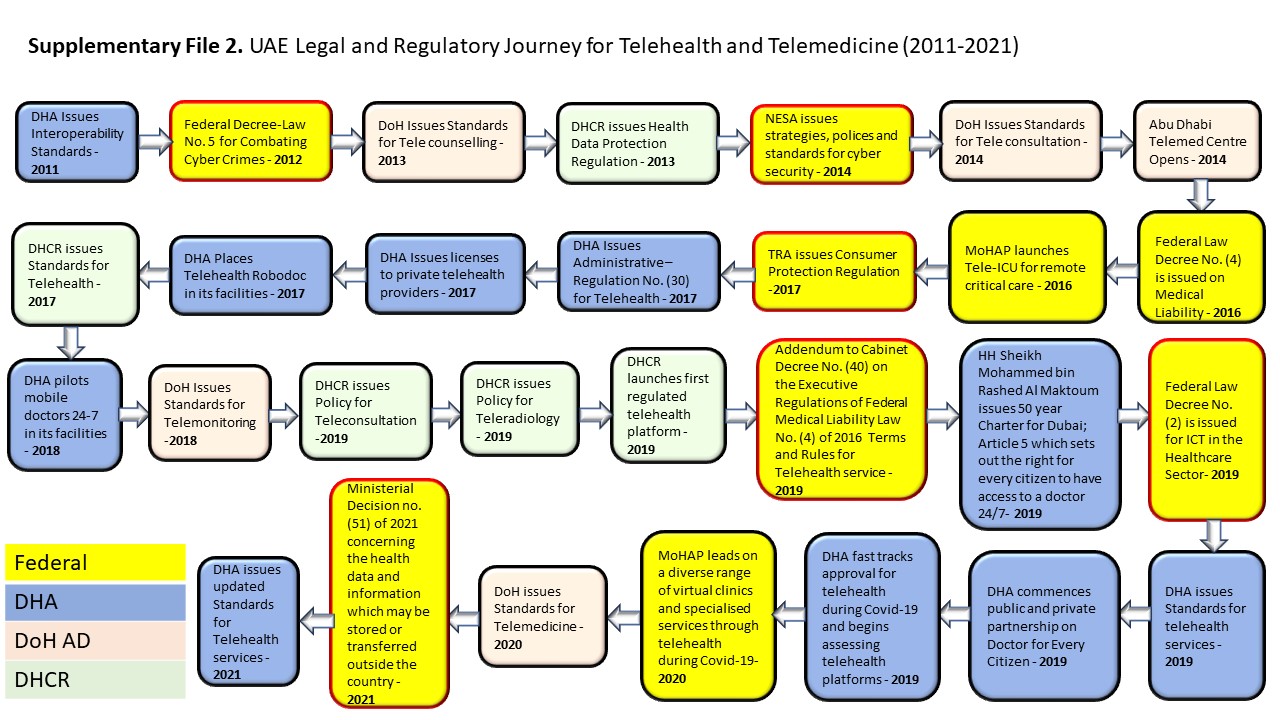

Supplement: sj-jpg-2-smo-10.1177_20503121221143223 – Supplemental material for Perspective: Telehealth – beyond legislation and regulation [file sj-jpg-2-smo-10.1177_20503121221143223.jpg]
